# Impact of cigarette price increase on health and financing outcomes in Vietnam

**DOI:** 10.12688/gatesopenres.13051.2

**Published:** 2020-04-28

**Authors:** Daphne C. Wu, Prabhat Jha, Sheila Dutta, Patricio Marquez

**Affiliations:** 1 Centre for Global Health Research, St. Michael's Hospital, Toronto, Toronto, ON, M5B 1W8, Canada; 2 World Bank Group Global Tobacco Program, World Bank, Washington, DC, 20433, USA

**Keywords:** Cigarette price, tobacco tax, extended cost-effectiveness analysis, tobacco economics, Vietnam, Southeast Asia

## Abstract

**Background:** Vietnam had about 15 million male smokers in 2015. To reduce adult tobacco use in Vietnam through an increase in the excise tax of cigarettes, we conducted an extended cost-effectiveness analysis to examine the impact of two scenarios of cigarette price increases.

**Methods:** We estimated, across income quintiles, the life-years gained, treatment cost averted, number of men avoiding catastrophic health expenditure and extreme poverty, and additional tax revenue under a 32% and a 62% increase in cigarette price through increased excise tax. We considered only male smokers as they constitute majority of the smokers. We used the average price elasticity of demand for cigarettes in Vietnam of -0.53.

**Results:** Under both scenarios of price increase, men in the poorest quintile would gain about 2.8 times the life-years and avert 2.5 times the treatment cost averted by the richest quintile. With a 32% price increase, about 285,000 men would avoid catastrophic health expenditure; as a result, about 95,000 men, more than half of whom in the poorest quintile, would avoid falling into extreme poverty. In contrast to the distribution of health benefits, the extra revenue generated from men in the richest quintile would be 1.2 times that from the poorest quintile. With a 62% price increase, about 553,000 men would avoid catastrophic health expenditure, and about 183,000 men, more than half of whom in the poorest quintile, would avoid falling into extreme poverty. The extra revenue generated from men in the richest quintile would be 3.8 times that from the poorest quintile.

**Conclusions:** Higher cigarette prices would particularly benefit the poorest income quintile of Vietnamese, in terms of health and financial outcomes. Thus, tobacco taxes are an effective way to improve health and reduce poverty in Vietnam.

## Introduction

According to the Global Adult Tobacco Survey (GATS), about 23.8% adults aged 15 years and older or about 15.3 million adults smoked tobacco in Vietnam in 2010 ^1^. About 97% of the cigarette smokers were males ^1^. By education level, the prevalence is highest among men with lower secondary and upper secondary education, and lowest among those with college education and higher (47% vs 30%). To reduce the prevalence of smoking, in 2013, the government of Vietnam launched the *National Strategy on Tobacco Control through 2020* with the target of reducing the rates of smoking from current prevalence by about 40% in youths aged 15–24 (target of 18% in 2020) and by about 20% for adults (target of 39% for men and <1% for women by 2020) ^2^. Vietnam was also one of the first countries in Asia to become a party of the World Health Organization Framework Convention on Tobacco Control (WHO FCTC) in 2005 ^2^. In 2012, the country also enacted the Law on Prevention and Control of Tobacco Harms, which significantly strengthened tobacco control policies. The new law established smoke-free places; increased the size of graphic health-warning labels; instituted a comprehensive ban on tobacco advertising, promotion, and sponsorship; and established the first Vietnam Tobacco Control Fund ^3^. Between 2010 and 2015, the prevalence of any tobacco smoking among males only fell from 47.4% to 45.3%; the prevalence among females remained at 1% ^1, 4^. The majority of the smokers smoke manufactured cigarettes (70%), with the remaining being smokers of traditional bamboo waterpipe (26%), hand-rolled cigarettes (2%), and other tobacco products (2%) ^4^.

Tobacco taxation is the single most effective intervention to increase cessation rates among current smokers and to decrease initiation by young people ^5^. According to Article 6 of the WHO FCTC, tobacco taxation policy is “an effective and important means of reducing tobacco consumption by various segments of the population, in particular young persons” ^6^. The Guidelines for Implementation of Article 6 of the WHO FCTC recognize that effective tobacco taxes significantly reduce tobacco consumption and prevalence ^6^. This consumption reduction role of tobacco taxation is due to the fact that special consumption or excise taxes increase prices of tobacco products relatively to other consumption products or income; and through this to reduce smokers’ demand. In order to do that, the Guidelines recommends that: “taxes rates should be monitored, increased or adjusted on a regular basis, potentially annually, taking into account inflation and income growth developments in order to reduce consumption of tobacco products” ^6^. Also, effective tobacco taxation may contribute significantly to state budgets, if increasing revenue growth outweighs the percentage decline in consumption of tobacco products.

### Taxation system on tobacco products in Vietnam

There are two types of excise taxes (called Special Consumption Tax (SCT)) in Vietnam: *Ad valorem* tax, which is levied as a percentage of the base price, and specific excise tax, which is levied as a specific value per unit of a product ^2^.

Immediately after the introduction of the SCT, cigarette and tobacco-product tax rates were differentiated, creating a complex system until 2005. Since 2005, the rates have been simplified and discrimination was gradually eliminated. Table 1 presents the evolution of SCT, and other taxes and tariffs on tobacco products since the inception of the SCT in 1990. In 1990, SCT rates on cigarettes were as follows: 50% of the factory price for filtered cigarettes and 40% of the factory price for non-filtered cigarette and cigars. 

**Table 1.** Evolution of Tobacco Special Consumption Tax, VAT and Import Tariffs. Values given as percentages.

Period	Special Consumption Tax (tax base is pre-tax factory price)				Value added tax	Tariffs
	Cigarettes			Cigars		
	Filtered produced from imported material	Filtered produced from domestic material	Non-filtered			
10/1990 – 8/1993	50	50	40	40	-	NA
9/1993 – 12/1995	70	52	32	32	-	NA
1/1996 – 12/1998	70	52	32	70	-	NA
1/1999 – 11/2001	65	45	25	65	-	NA
11/2001 – 12/2003	65	45	25	65	-	Import prohibited
1/2004 – 12/2005	65	45	25	65	10	Import prohibited
1/2006 – 12/2006	55			65	10	Import prohibited
1/2007 – 12/2007	55			65	10	100
1/2008 – 12/2009	65				10	140
1/2009 – 12/2015	65				10	140
1/2016 – 12/2017	70	70	70	70	10	135
1/2018 –	75	75	75	75	10	135

NA: Not available. Sources: Tax Policy Department (TPD) – Ministry of Finance (MOF).

The lower tax rates on cigarettes manufactured with domestic raw materials favoured domestic brands and encouraged per adult consumption of cigarettes. As with many tax policies in transition economies, this policy was intended to support domestic tobacco cultivation while discouraging imports of raw materials, and to increase state revenues from smokers of cigarettes made from imported raw materials, who had higher income. In retrospect, these were likely unwise choices as the short term demand generation has only limited impact on the medium term supply of raw tobacco, and because the tax strategy effectively made cheaper cigarettes available that were taken up most by the poor.

However, in order to meet the requirements to join the World Trade Organization (WTO), in 2005, the National Assembly amended the SCT and approved a new VAT (Value Added Tax) Law. Under this amendment, from 2006, cigarettes were taxed at 55% and were subsequently increased to 65% in 2008. The implementation of non-discriminatory tax rates was a step forward for Vietnam’s international integration policy, although those excise rates were *ad valorem* rather than, specific, as recommended in World Bank ^7^.

Following the development of new tobacco products, the SCT Tax Law amendment at the end of 2008 subjected other tobacco products (used for chewing especially) to an unchanged excise tax rate of 65%.

VAT was introduced in the last decade and was part of the country’s tax modernization. All organizations and individuals engaging in manufacturing and conducting business in tobacco or importing tobacco are required to pay taxes. The VAT taxable price of cigarettes sold or supplied by production or business establishments is the sales price including the excise tax but excluding VAT. For imported tobacco, the VAT taxable price is the import price at the border gate plus import duties and plus excise tax. VAT rates on tobacco products were uniform and maintained constant. In Vietnam, there are two overall VAT rates, a standard 10% which is applied to most goods and services and a reduced 5% which is applied to basic foods, transport, medical equipment, and agricultural production and services. Tobacco falls in the 10% category. The rate is zero, though, for exports of tobacco products, as for all exports, and VAT paid for inputs of goods and services is refunded.

### Current tobacco tax structure and rates in Vietnam

- SCT: Vietnam levies a uniform *ad valorem* excise tax on all cigarettes ^8^. The SCT was unified for all tobacco products from 2006. Since January 2019, the tax rate was 75%. For domestic tobacco products, the tax base is the factory price (without VAT and excise tax).
- VAT: The current VAT rate is 10%, and for domestic tobacco products, the tax base is factory price plus the excise tax.
- Tariff: The current tariff rate for cigarettes is 135%. The tax base for import tax is the import (CIF) price.
- Tobacco Control Fund: The Tobacco Control Fund (TCF) was established in 2012 under the Vietnam Tobacco Control Law. The TCF receives a compulsory contribution of 1% of the factory price of all cigarette packs produced locally or imported for local consumption beginning from May 2013. This rate was increased to 2% from May 2019 ^9^.

The proposed plan to raise tobacco taxes was submitted in August 2017. The draft law suggested amending and supplementing some articles of the Law on the Value Added Tax, the Law on Special Consumption Tax, the Law on the Corporate Income Tax, and the Law on Personal Income Tax. Regarding the tobacco excise tax, it was proposed to apply the mixed excise tax, in addition to the current tax starting from January 1, 2020. This is to be done by either applying a specific tax of Vietnamese Dong (VND) 1,000 per pack of 20 cigarettes ^2^, or by increasing the *ad valorem* tax from 75% to 80% of the tobacco’s price from 2020 onwards and from 80% to 85% from 2021 onwards ^10^. Health officials favour the first option, but strongly argued that the fixed (specific) tax be higher at VND2,000–5,000 ^11^.

This paper is part of additional efforts supported by the World Bank Global Tobacco Control Program to inform the Government of Vietnam on options for tobacco taxation by providing estimates of the impact of cigarette price increase across five income groups for the period 2020–2022 under two scenarios:

- Scenario A: Increase in *ad valorem* tax from the current 75% to 90%, plus an introduction of a specific tax at VND3,000 per pack of 20 cigarettes, which, taken together, constitutes a 32% increase in price.
- Scenario B- Increase in *ad valorem* tax from the current 75% to 120%, plus an introduction of a specific tax at VND5,000 per pack of 20 cigarettes, which, taken together, constitutes a 62% increase in price.

The two scenarios of tax increase were proposed by the Government of Vietnam.

## Methods

We used the model from the Disease Control Priorities Project which draws upon the analytic framework of the Asian Development Bank to estimate the fiscal, health and poverty impact of increasing cigarette taxes ^12, 13^. The model was previously used by the Global Tobacco Economics Consortium (GTEC) to estimate the impact of a 50% increase in the price of cigarettes on health, poverty, and financial outcomes in 13 middle-income countries ^5^.

### Study population

We focussed on male cigarette smokers aged 15 years and older, as males comprised the vast majority of cigarette smokers in Vietnam (about 12.1 million out of 12.4 million cigarette smokers overall, or 98% in 2015; about 3.1 million men smoked hand-rolled tobacco, traditional bamboo waterpipe, shisha waterpipe, pipe, cigars/cheroots/cigarillos, and other forms of smoking tobacco). To estimate the number of smokers by age and income groups, we applied the age-specific smoking prevalence for males from the GATS survey conducted in Vietnam in 2015 to the number of males in each age group in 2017 ^4^. We estimated the population in each age group by applying the proportion of male population in each age group from the 2009 census of Vietnam to the male population in Vietnam in 2017 obtained from the General Statistics Office of Vietnam ^14, 15^. As the GATS survey did not collect information on household income, we used education level as a proxy measure of income group, as the Vietnam National Health Survey 2001 showed that prevalence of tobacco use among males is similar when classified by income quintiles and education levels ^16^. We applied the relative prevalence of smoking among illiterate males, and those with completed primary, lower secondary, upper secondary, and college education to the number of smokers in each age group to obtain the number of smokers in each age and income group.

### Cigarette price and price increase

The market price of cigarettes used was that of Vinataba, the most-sold brand of cigarettes in Vietnam, as obtained from the World Health Organization Report on the Global Tobacco Epidemic 2017 ^17^. The same source was used to obtain the *ad valorem* tax and VAT, as percentage of the final retail price. Using the current factory price of VND8,028 calculated by Fuchs and colleagues using the market price of VND20,000, *ad valorem* rate of 75%, VAT rate of 10% and mandatory contribution to the Tobacco Control Fund of 2%, as per the current tax structure, we calculated the percentage increase in the retail price under two scenarios:

- Scenario A: Increase in *ad valorem* tax from the current 75% to 90%, plus an introduction of a specific tax at VND3,000 per pack of 20 cigarettes (corresponding to a retail price increase of 32%), and
- Scenario B: Increase in *ad valorem* tax from the current 75% to 120%, plus an introduction of a specific tax at VND5,000 per pack of 20 cigarettes (corresponding to a retail price increase of 62%).

We assume, realistically, that the tax increases will be passed on to consumer prices. The industry can delay passing them fully through in the short term but will not do so at the expense of their profit margins for any reasonable time. Indeed, recent analyses of modest tax hikes and responsiveness across the states of India showed that nearly all tax hikes were more than passed onto smokers (i.e., small tax hikes enabled rent-seeking opportunities by the cigarette industry), but the few tax decreases did not reduce consumer prices; ^18^ this is consistent with the profit-maximising behaviour of the tobacco industry.

### Price effects on smoking

To estimate the number of smokers who would quit as a result of the price increase, we used the estimated price elasticity for cigarette demand in Vietnam of -0.53 ^19^. As young people and those on low income shower greater price sensitivity ^20, 21^, we used two times the national elasticity for young smokers (15–24 years) and applied this higher price elasticity to future smokers (those below 15 years) who have not yet started to smoke, as done previously by GTEC ^5^. For those in the bottom (poorest 20% of the population) and those in the top income group (richest 20% of the population), we used the price elasticity reported by Kinh and colleagues (2006) for those in the low income quintile and high income quintile in Vietnam of -0.85 and -0.35 respectively ^22^. We assumed price elasticities of quitting at half of the price-elasticity of cigarette demand ^5^.

### Effects of cigarette price increase on life-years gained, disease costs, income poverty, and taxes paid

We followed the methodology of the previous analysis of GTEC to estimate the impact of a cigarette price increase on number of deaths averted due to four major tobacco-attributable diseases (chronic obstructive respiratory disease (COPD), stroke, heart disease and cancer), life-years gained, treatment cost averted due to the four tobacco-attributable diseases, number of men avoiding catastrophic health expenditures and extreme poverty, and additional tax revenues collected ^5^. The treatment cost for COPD, stroke, heart disease and cancer were obtained from the Statistics Yearbook of Vietnam 2011 ^23^. The average income in each income quintile was obtained from Statistical Yearbook of Vietnam 2016 ^24^. All costs and prices were converted into International dollars ($Int, which convert local currencies at exchange rates that account for differences in Purchasing Power Parity). We adjusted the International dollars for inflation using consumer price index and exchange rates obtained from the World Bank Development Indicators ^25^.

The data inputs and sources of data are available as *Underlying data* ^26^.

### Sensitivity analysis

We conducted sensitivity analyses to examine the impact of a 25%, 50%, and 100% price increase with the cigarette price elasticity of demand in Vietnam of -0.53, and the impact of a 32% (Scenario A) and 62% (Scenario B) price increase with the average price elasticity of demand for cigarettes in both high income, and low- and middle-income countries of -0.40 (universal elasticity) ^20, 27^. For those on low income, we used a price elasticity of -0.635, as done by GTEC ^5^.

## Results

Before the cigarette price increase, an estimated total of about 12.1 million males aged 15 years and older smoked cigarettes in Vietnam ( Table 2). Men in the bottom income group constitute about 18%, while men in the top income group constitute about 16% of the total number of male smokers. This is a small difference across income groups by international standards. Men in the lower-middle and middle income groups account for about 50% of the total number smokers.

**Table 2.** Impact of cigarette price increase on quitting, deaths averted and life-years gained in Vietnam.

Variables by income groups	Scenario A: 32% price increase * ^‡^	Scenario B: 62% price increase ^†‡^
**Number of male smokers aged ≥15 years** **before price increase (in millions)**		
First (bottom 20%)	2.2	
Second	3.0	
Third	3.0	
Fourth	1.9	
Fifth (top 20%)	1.9	
Total	12.1	
First: fifth ratio	1.2	
**Number of men who quit smoking after tax** **increase (in thousands)**		
First (bottom 20%)	376.6	729.7
Second	433.7	840.3
Third	358.3	694.1
Fourth	183.5	355.6
Fifth (top 20%)	132.9	257.5
Total	1,485.0	2,877.2
First: fifth ratio	2.8	2.8
**Total deaths averted due to COPD, stroke,** **heart disease, and cancer (in thousands)**		
First (bottom 20%)	159.7	309.4
Second	183.9	356.2
Third	151.9	294.3
Fourth	77.8	150.7
Fifth (top 20%)	56.3	109.2
Total	629.6	1,219.8
First: fifth ratio	2.8	2.8
**Total life-years gained (in millions)**		
First (bottom 20%)	2.8	5.4
Second	3.2	6.2
Third	2.6	5.1
Fourth	1.4	2.6
Fifth (top 20%)	1.0	1.9
Total	10.1	21.3
First: fifth ratio	2.8	2.8

*Scenario A- Increase in *ad valorem* tax from the current 75% to 90% plus an introduction of a specific tax at VND3,000 per pack (equivalent to 32% increase in retail price).

^†^ Scenario B- Increase in *ad valorem* tax from the current 75% to 120% plus an introduction of a specific tax at VND5,000 per pack (equivalent to 62% increase in retail price).

^‡^ Price elasticity used, by income group: First -0.85, second/third/fourth -0.53, fifth -0.35.

## Impact of cigarette price increase under Scenario A

An increase in cigarette price under Scenario A, which would be equivalent of a 32% increase in the retail price, would lead to about 1,485,000 men quitting smoking, with the bottom income group having 2.8 times as many quitters as the top income group (377,000 vs 133,000) ( Table 2). An estimated total of 630,000 deaths due to COPD, stroke, heart disease, and cancer would be averted among current smokers due to quitting. The number of averted deaths in the bottom income group would be 2.8 times that in the top income group (160,000 vs 56,000). The deaths averted due to quitting would yield about 11 million life-years, with the bottom income group gaining 2.8 times more life-years than those the top income group (2.8 million vs 1 million). In absolute terms, over a quarter of the overall reduced deaths and life years gained would occur in the lowest income group of men.

The cost averted for treating the four major tobacco-attributable diseases would amount to about VND9,746 billion ($Int 1.3 billion) ( Table 3). The treatment cost – and suffering – averted in the bottom income group would be 2.5 times higher than in the top income group (VND2,346 billion vs 949 billion, or $Int 304 million vs 123 million). About 285,000 men would avoid catastrophic health expenditures, with the number of men in the bottom income group being 5.5 times that in the top income group (73,000 vs 13,000). As a result of the catastrophic health expenditures averted, about 94,500 men would avoid falling into extreme poverty as defined by the World Bank as income of under $1.90 per day in purchasing power parity. The number of families falling into extreme poverty would be somewhat smaller, depending on earnings by other household members, but would still be large. The increase in excise tax needed to achieve the cigarette price increase would generate more than VND11.7 trillion ($Int 1.5 billion). In contrast to the distribution of health benefits, the extra revenue generated from men in the top income group would be a modest 1.2 times that from the bottom income group (VND2 trillion vs 1.7 trillion, or $Int 264 million vs 225 million).

**Table 3.** Impact of cigarette price increase in Vietnam under Scenario A and Scenario B on treatment cost averted, number of men avoiding catastrophic health expenditures and extreme poverty, and additional tax revenue collected in Vietnam.

Variables by income groups	Scenario A: 32% price increase * ^‡^	Scenario B: 62% price increase ^†‡^
**Treatment cost averted (in LCU, billions ($Int,** **millions))**		
First (bottom 20%)	2,346 (304)	4,545 (589)
Second	2,901 (376)	5,618 (728)
Third	2,323 (301)	4,506 (584)
Fourth	1,227 (159)	2,377 (308)
Fifth (top 20%)	949 (123)	1,837 (238)
Total	9,746 (1,263)	18,882 (2,447)
First: fifth ratio	2.5	2.5
**Number of men avoiding catastrophic health** **expenditures (in thousands)**		
First (bottom 20%)	72.6	140.7
Second	89.7	173.8
Third	72.0	139.4
Fourth	38.0	73.6
Fifth (top 20%)	12.9	25.0
Total	285.2	552.5
First: fifth ratio	5.6	5.6
**Number of men avoiding extreme poverty**		
First (bottom 20%)	72,621	140,704
Second	12,124	23,491
Third	9,734	18,841
Fourth	0	0
Fifth (top 20%)	0	0
Total	94,479	183,036
First: fifth ratio	-	-
**Additional tax revenues (in LCU, billions ($Int,** **millions))**		
First (bottom 20%)	1,737 (225)	827 (107)
Second	2,780 (360)	2,444 (317)
Third	3,059 (396)	3,556 (461)
Fourth	2,149 (279)	2,955 (383)
Fifth (top 20%)	2,039 (264)	3,137 (406)
Total	11,764 (1,525)	12,918 (1,674)
First: fifth ratio	0.85	0.26

*Scenario A- Increase in *ad valorem* tax from the current 75% to 90% plus an introduction of a specific tax at VND3,000 per pack (equivalent to 32% increase in retail price).

^†^Scenario B- Increase in *ad valorem* tax from the current 75% to 120% plus an introduction of a specific tax at VND5,000 per pack (equivalent to 62% increase in retail price).

^‡^ Price elasticity used, by income group: First -0.85, second/third/fourth -0.53, fifth -0.35.

## Impact of cigarette price increase under Scenario B

A cigarette price increase under Scenario B which is an equivalent of a 62% increase in the retail price of cigarettes would result in about 2,877,000 men quitting smoking. Of this, the bottom income group will have 2.8 times as many quitters as the top income group (730,000 vs 258,000) ( Table 2). Quitting as a result of the price increase would avert about 1.2 million deaths due to COPD, stroke, heart disease, and cancer among male smokers. The number of deaths averted in the bottom income group would be 2.8 times that in the top income group (309,000 vs 109,000). As a result of the deaths averted, Vietnam would gain about 21 million life-years and avert about VND 18,882 billion ($Int 2.4 billion) in treatment cost for treating the four major tobacco-attributable diseases ( Table 3). The averted treatment cost in the bottom income group would be about 2.5 times that in the top income group (VND4,545 billion vs 1,837 billion, $Int 589 million vs 238 million). About 552,000 men would avoid catastrophic health expenditures, with the bottom income group avoiding 5.6 times that of the top income group (141,000 vs 25,000). As a result of the catastrophic health expenditures averted, about 183,000 men would avoid falling into extreme poverty. The tax increase would generate about VND12.9 trillion ($Int 1.7 billion), with contribution from the top income group being about 4 times that from the bottom income group (VND3,137 billion vs VND827 billion, $Int 407 million vs $Int 104 million). The extra tax revenue is particularly progressive in this scenario of a 62% price increase than the smaller increase.

## Comparison of 50% price increase in Vietnam vs in Indonesia

To compare the impact of cigarette price increase in Vietnam vs in other Southeast Asian countries, we used the findings of GTEC (2018). Table 4 shows the impact of a 50% cigarette price increase on the number of males who quit after the price increase, deaths averted, life-years gained, treatment cost averted, number of men avoiding catastrophic health expenditures and extreme poverty, and the additional tax revenue collected in Vietnam and Indonesia, according to GTEC (2018). Compared to Vietnam, with a 50% cigarette price increase, the ratio of the number of quitters, tobacco-attributable deaths averted, and life-years gained between the bottom and the top income group is greater in Indonesia. However, the ratio of the number of men avoiding extreme poverty is substantially higher in Vietnam than in Indonesia.

**Table 4.** Cumulative impact of a 50% cigarette price increase in Vietnam and Indonesia (from GTEC, 2018) ^5^.

	Vietnam ^§^	Indonesia ^§^
**Number of male smokers aged ≥15 years before** **price increase (in millions)**		
First (bottom 20%)	3.7	13.6
Second	3.3	12.0
Third	2.6	9.8
Fourth	2.6	9.7
Fifth (top 20%)	2.2	7.7
Total	13.2 ^¶^	52.9
First: fifth ratio	1.5	1.8
**Number of men who quit smoking after price** **increase (in thousands)**		
First (bottom 20%)	785.8	3,255.5
Second	569.6	2,292.4
Third	336.1	1,406.3
Fourth	214.9	915.4
Fifth (top 20%)	99.8	357.4
Total	2,006.1	8,227.0
First: fifth ratio	7.9	9.1
**Total deaths averted due to COPD, stroke, heart** **disease, and cancer (in thousands)**		
First (bottom 20%)	341.6	1,418
Second	259.1	998
Third	179.0	612
Fourth	112.9	399
Fifth (top 20%)	49.4	156
Total	941.9	3,582
First: fifth ratio	6.9	9.1
**Total life-years gained (in millions)**		
First (bottom 20%)	5.6	22.5
Second	4.1	15.8
Third	2.4	9.7
Fourth	1.5	6.3
Fifth (top 20%)	0.7	2.5
Total	14.3	56.8
First: fifth ratio	7.9	9.1
**Treatment cost averted (in LCU, billions ($Int,** **millions))**		
First (bottom 20%)	2,284 (296)	19,776 (4,120)
Second	1,798 (233)	15,456 (3,220)
Third	1,536 (199)	13,296 (2,770)
Fourth	910 (118)	10,512 (2,190)
Fifth (top 20%)	564 (73)	5,040 (1,050)
Total	7,092 (919)	60,080(13,350)
First: fifth ratio	4.0	3.9
**Number of men avoiding catastrophic health** **expenditures (in thousands)**		
First (bottom 20%)	112.6	637.9
Second	88.6	499.1
Third	75.7	428.6
Fourth	44.9	338.8
Fifth (top 20%)	27.7	163.4
Total	349.6	2,067.9
First: fifth ratio	4.1	3.9
**Number of men avoiding extreme poverty**		
First (bottom 20%)	107,418	594,663
Second	77,294	499,090
Third	14,323	426,300
Fourth	3,790	84,068
Fifth (top 20%)	203	20,110
Total	203,028	1,624,2231
First: fifth ratio	529.1	29.6
**Additional tax revenues (in LCU, billions ($Int,** **millions))**		
First (bottom 20%)	4,213 (546)	323 (67)
Second	3,464 (449)	806 (168)
Third	3,395 (440)	1,181 (246)
Fourth	3,935 (510)	1,661 (346)
Fifth (top 20%)	3,734 (484)	2,155 (449)
Total	18,743 (2,429)	6127 (1,280)
First: fifth ratio	1.1	0.2

^§^Price elasticity used, by income group: First -0.635, second/third/fourth -0.4, fifth -0.122.

^¶^Number of male smokers aged ≥15 years in Vietnam is higher in the GTEC analysis than in our current analysis because the GTEC analysis used the male smoking prevalence in 2010 which is higher than the prevalence in 2015 used in our current analysis (overall male smoking prevalence: 39.1% vs 36.1%).

## Sensitivity analysis

**Figure 1.**
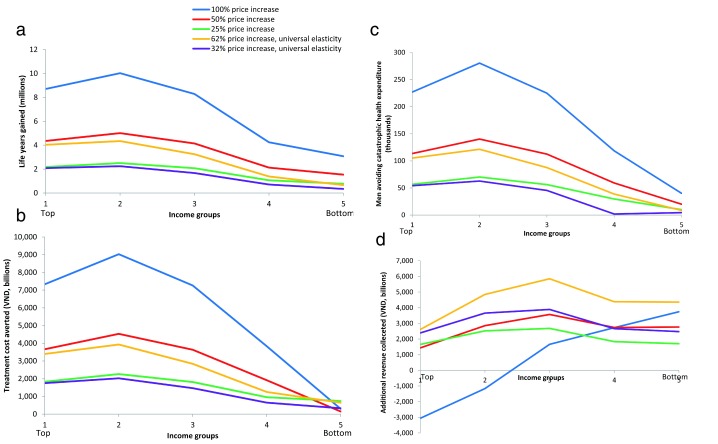
Sensitivity analysis. Shown are analyses for the impact of a 25%, 50%, and 100% price increase with -0.53 price elasticity (cigarette price elasticity of demand in Vietnam), and for a 32% and 62% price increase with the universal price elasticity of -0.40 on ( **a**) life years gained, ( **b**) treatment cost averted, ( **c**) number of men avoiding catastrophic health expenditure, ( **d**) additional revenue collected.

Figures 1a–c shows the result of our sensitivity analyses of the impact of varying levels of price increase and using the universal price elasticity of -0.40 on life-years gained, treatment costs averted and catastrophic health expenditures avoided, respectively. Using the price elasticity in Vietnam (-0.53), with a 25%, 50% and 100% price increase, the ratio of the number of life-years gained between the bottom and the top income groups is 2.8 for all price increases ( Figure 1a). The ratio increases to 6.1 when we apply the universal price elasticity to a price increase of 32% and 62%. Similarly, when the price elasticity is -0.40, the ratio of the treatment cost averted and catastrophic health expenditures avoided by the bottom versus the top income group for all price increases, except for treatment cost averted with 100% price increase, is 2.5 and 5.6 respectively, and increases to 5.3 and 12.1 respectively, when the price elasticity is -0.40 ( Figure 1b, c). The additional tax revenue collected from the top income group with a 50% and a 25% price increase with -0.53 price elasticity and a 62% and a 32% price increase with -0.40 price elasticity is between 1 and 2 times that of the bottom income group ( Figure 1d). With a 100% price increase, about 95% of the tax burden would be borne by the top income group. 

## Discussion

We found that a cigarette price of 32% or 62% in Vietnam would favour the bottom income group of the population more strongly in terms of deaths averted, life-years saved, out-of-pocket expenditures for treating tobacco-attributable diseases, catastrophic health expenditures, and extreme poverty averted. Our findings are consistent with the earlier findings of GTEC of the impact of a 50% increase in cigarette price in 13 middle-income countries, which challenges the conventional view that tobacco taxes are more detrimental to people on low versus high income ^5^.

Tobacco tax hikes in other countries have shown that when taxes increase, consumption decreases and smoking rates decrease, but government revenue still rises. Jha and colleagues recently showed that higher cigarette prices substantially reduced smoking, even after accounting for illegal cigarette sales, in France and Canada ^28^. In Canada, when tobacco tax was lowered in the early 1990s in response to illicit tobacco trade instigated by the tobacco industry, consumption rose. In Thailand, between 1993 and 2012, the SCT on cigarettes was increased 10 separate times, about one tax increase every two years, from 120% to 670% of the factory price ^2^. As a result of the price increase, the smoking prevalence decreased from 32% in 1991 to 20% in 2015, while tobacco tax revenue increased more than four times ^2^. The tax increase also did not lead to smuggling, as GATS 2011 in Thailand showed that only 4.8% of smokers used smuggled cigarettes ^29^. In the Philippines, prior to 2012, a four-tiered excise tax system, with various tax rates ranging from 2.72 Philippine Pesos (PHP) to 28.3 PHP per pack of cigarettes as applicable to tobacco products at different prices was used ^2^. In 2012, the four-tiered tax structure was replaced by a two-tiered tax structure and tax rates on cigarettes was steadily increased from 2013 to 2016, reaching a common tax rate of 30 PHP per pack in 2017 ^2^. As a result of the tax increase, the rate of smoking among adults fell from 30% in 2009 to 24% in 2015, ^30^, while the tobacco tax revenue increased by more than three times ^2^. Compared to Thailand and the Philippines, Vietnam has the highest annual consumption of 3,900 million packs but collects only about one-third of the total tobacco tax collected in Thailand or the Philippines due to the current low tax rate. Increase in tobacco taxes could generate substantial revenues that could be used to finance universal health coverage in Vietnam. Although the tax revenue itself would not provide enough to meet the financial needs of universal health coverage, it would make a significant contribution ^5^.

Our study has some limitations, including the assumptions of price elasticities. Variation in price responsiveness has been reported in Vietnam, as recently reviewed by Fuchs and colleagues ^31^. However, various sensitivity analyses suggested that variation in elasticity did not influence the overall conclusions greatly. Second, our study used the price of the most-sold brand of cigarettes, Vinabata, which comprised about 60% of the market share in 2015–2017 ^32^. The average cigarette price is much lower than the price of the most-sold brand. The average cigarette price was VND15,000 per pack and the price of Vinabata was VND20,200 per pack in 2017 ^33^. The Vietnam tax structure, with much higher taxes per cigarette on higher price brands, encourages downward substitution between brands, reducing quitting and decreased consumption. A set of recent analyses has shown that tobacco tax increase needs to be substantial so as to avoid downward substitution and prevent the rent seeking opportunities by the cigarette industry ^7^. A large tax hike means greater revenue generation for the government versus profits for the industry. A key argument of this analysis is that to in order to maximize the health benefits, large increases in tax should preferentially be imposed on the cheapest brands. This is quite consistent with the policy guidance from the World Bank to move to specific (rather than *ad valorem*) taxes that are equal across cigarette price categories ^7^. Reassuringly, the overall results focused here on avoidance of out of pocket expenditures are consistent with a recent analysis by Fuchs and colleagues that focused on net income gains across declines in Vietnam smokers ^31^. The main differences in details arise in taking the male smoker as the unit of analysis here as compared to the household in Fuchs and colleagues’ study, as well as in other minor differences in the methodologies applied. Third, we used education level as a proxy indicator for income quintile. Due to data limitations, we could not verify whether the income used to represent each quintile corresponds to the income by education level. Fourth, our model applied only to the entire lifetime of the current cohort of smokers, hence it underestimates the effects on future consumption decreases, particularly if the large early price hikes also lead to higher future price expectations ^5^. Finally, the assumption that the poor are more price responsive was central to our analysis, and while the exact responsiveness to price does likely vary in Vietnam from other settings, there is substantial earlier evidence, in Vietnam as well as globally, to document that the poor are in fact more responsive to price ^31^.

## Conclusions

Vietnam has made substantial progress in reducing tobacco use. Further progress is likely to be possible with large increases in price, particularly those that focus on narrowing the gap between the least and most expensive cigarettes. Higher cigarette taxes would also reduce poverty by reducing out of pocket health expenditures among the poorest smokers.

## Data availability

### Underlying data

Figshare: Data input and data sources for extended cost-effectiveness analysis of cigarette price increase in Vietnam, https://doi.org/10.6084/m9.figshare.9033914 ^34^.

This project contains the pooled data used in the present study, alongside the original source of the data. 

This file is available under the terms of the Creative Commons Attribution 4.0 International license (CC-BY 4.0).
