# Supplementary material for: Impact of cigarette price increase on health and financing outcomes in Vietnam
Source: Gates Open Res. 2020 Apr 28;3:1516. Originally published 2019 Aug 14. [Version 2] doi: 10.12688/gatesopenres.13051.2 (PMC7216405; doi:10.12688/gatesopenres.13051.2)
Supplement: Peer review history

# Peer review: Reviewer response for version 2

**DOI:** 10.21956/gatesopenres.14315.r28809

**Published:** 2020-05-20

**Authors:** Alfred Kechia Mukong

**Affiliations:** 1 Department of Economics, University of Namibia, Windhoek, Namibia

I have reviewed their response to my comments and I am satisfied. I don't have further comments for this paper.

Is the work clearly and accurately presented and does it cite the current literature?

Yes

If applicable, is the statistical analysis and its interpretation appropriate?

Partly

Are all the source data underlying the results available to ensure full reproducibility?

Yes

Is the study design appropriate and is the work technically sound?

Partly

Are the conclusions drawn adequately supported by the results?

Yes

Are sufficient details of methods and analysis provided to allow replication by others?

Partly

Reviewer Expertise:

NA

I confirm that I have read this submission and believe that I have an appropriate level of expertise to confirm that it is of an acceptable scientific standard.

# Peer review: Reviewer response for version 2

**DOI:** 10.21956/gatesopenres.14315.r28807

**Published:** 2020-05-11

**Authors:** Guillermo Paraje

**Affiliations:** 1 Business School, Universidad Adolfo Ibáñez, Santiago, Chile

I am happy with the new version.

Is the work clearly and accurately presented and does it cite the current literature?

Yes

If applicable, is the statistical analysis and its interpretation appropriate?

Yes

Are all the source data underlying the results available to ensure full reproducibility?

Yes

Is the study design appropriate and is the work technically sound?

Partly

Are the conclusions drawn adequately supported by the results?

Partly

Are sufficient details of methods and analysis provided to allow replication by others?

Partly

Reviewer Expertise:

Economics; Development Economics; Health Economics

I confirm that I have read this submission and believe that I have an appropriate level of expertise to confirm that it is of an acceptable scientific standard.

# Peer review: Reviewer response for version 1

**DOI:** 10.21956/gatesopenres.14178.r27815

**Published:** 2019-11-01

**Authors:** Alfred Kechia Mukong

**Affiliations:** 1 Department of Economics, University of Namibia, Windhoek, Namibia

This paper estimates across income quintiles the life-years gained, treatment cost averted, number of men avoiding catastrophic health expenditure and extreme poverty, and additional tax revenue for 32% and a 62% increase in cigarette prices using an extended cost-effectiveness analysis. The authors showed that under both scenarios of price increase, men in the poorest quintile would again about 2.8 times the life-years and avert 2.5 times the treatment cost averted by the richest men. Poorest men would equally avoid catastrophic health expenditure and falling into extreme poverty but extra revenue is generated from the richest than from the poorest quintile. However, there is room to improve on the analysis if the following comments and concerns are addressed. 

- Using the overall average price elasticity of demand for cigarettes across the income quintile reduces the validity for comparing the estimates across the different income quintiles. It is evidence that the price elasticity of demand for cigarettes is generally different across the different income quintile. As a solution, you either estimate price elasticities by income quintile or take average elasticity for each quantile, if such elasticities exist for Vietnam.
- The authors highlighted that the paper is part of the effort of the World Bank Global Tobacco Control Program to inform the government of Vietnam on the estimated impact of cigarette price increase across five income groups. Assuming demands elasticities are equal across the five groups when they are not in really therefore provide misleading estimates. The authors need to think critically on how to address this problem.
- Another challenge is assuming levels of educations as proxies for income groupings. The fact that the prevalence of tobacco use is similar when classified by income quintiles and education levels, does not mean the level of responsive to price will be similar too. If this is the case then education level will not be a good proxy for income group, hence the estimates in the paper are far from valid. Is it possible to compute price elasticity by educational attainment in Vietnam for consistency of the analysis in this paper (since education level is used as a proxy for income)?
- Another concern is the use of the price of the most-sold brand of cigarettes in Vietnam. What share of the market for cigarette does this brand occupy? If this is less than 80%, then the average price of cigarettes sold in Vietnam will be more appropriate than the price of the most sold brand. If the price most-sold brand of cigarettes is not the lowest, such a policy change may see consumers moving for the high priced brand to the low priced brands (not necessary reducing or quitting or reducing consumption of cigarettes).
- The average income in each quintile is used and obtained from Statistical Yearbook of Vietnam 2016. If education level is used as a proxy for income quintile, then for consistency, the income for each education level should be considered.
- The paper assumes two scenarios – scenario A considers a 32% increase in price and scenario B considers a 62% increase in price. However, there are no discussions to justify the choice of the different scenarios. If it is what the government of Vietnam intend to do then the authors need to clearly state this in the work.
- In the abstract, it is stated that Vietnam has over 15 million male smokers but in the study population section, the authors argued that their focus is on male smokers aged 15 years and older, because males comprised the vast majority of cigarette smokers in Vietnam (about 12.1 million out of 12.4 million overall). There is not information about the 2.9 million of the 15 million. What do the smoke? Or are they less than 15 years. This need to be made clear.

Is the work clearly and accurately presented and does it cite the current literature?

Yes

If applicable, is the statistical analysis and its interpretation appropriate?

Partly

Are all the source data underlying the results available to ensure full reproducibility?

Yes

Is the study design appropriate and is the work technically sound?

Partly

Are the conclusions drawn adequately supported by the results?

Yes

Are sufficient details of methods and analysis provided to allow replication by others?

Partly

Reviewer Expertise:

NA

I confirm that I have read this submission and believe that I have an appropriate level of expertise to confirm that it is of an acceptable scientific standard, however I have significant reservations, as outlined above.

# Response

**Published:** 2020-04-16

**Authors:** Daphne C. Wu

**Affiliations:** Centre for Global Health Research, Canada

1. Using the overall average price elasticity of demand for cigarettes across the income quintile reduces the validity for comparing the estimates across the different income quintiles. It is evidence that the price elasticity of demand for cigarettes is generally different across the different income quintile. As a solution, you either estimate price elasticities by income quintile or take average elasticity for each quantile, if such elasticities exist for Vietnam. *Response*- For those in the bottom and top income groups, we used the price elasticities of -0.85 and -0.35 respectively, as reported by Kinh and colleagues in Vietnam (please see page 5).
2. The authors highlighted that the paper is part of the effort of the World Bank Global Tobacco Control Program to inform the government of Vietnam on the estimated impact of cigarette price increase across five income groups. Assuming demands elasticities are equal across the five groups when they are not in really therefore provide misleading estimates. The authors need to think critically on how to address this problem. *Response-* We used different price elasticities for those in the top and bottom income quintile (please see our response to comment 1 above). 
3. Another challenge is assuming levels of educations as proxies for income groupings. The fact that the prevalence of tobacco use is similar when classified by income quintiles and education levels, does not mean the level of responsive to price will be similar too. If this is the case then education level will not be a good proxy for income group, hence the estimates in the paper are far from valid. Is it possible to compute price elasticity by educational attainment in Vietnam for consistency of the analysis in this paper (since education level is used as a proxy for income)? *Response- *As mentioned in the Discussion section, one of the limitations of our study is the assumption on price elasticity. Most studies examining the price elasticity of cigarette demand in Vietnam reports the price elasticity by income groups, and due to data limitations, we are not able to compute the price elasticity by education level. However, as our sensitivity analysis shows, variation in elasticity did not significantly change our overall conclusions.
4. Another concern is the use of the price of the most-sold brand of cigarettes in Vietnam. What share of the market for cigarette does this brand occupy? If this is less than 80%, then the average price of cigarettes sold in Vietnam will be more appropriate than the price of the most sold brand. If the price most-sold brand of cigarettes is not the lowest, such a policy change may see consumers moving for the high priced brand to the low priced brands (not necessary reducing or quitting or reducing consumption of cigarettes). *Response-* We have added this as a limitation of our study.
5. The average income in each quintile is used and obtained from Statistical Yearbook of Vietnam 2016. If education level is used as a proxy for income quintile, then for consistency, the income for each education level should be considered. *Response- *Due to data limitations, we could not obtain the income by education level. We have added this as a limitation of our study.
6. The paper assumes two scenarios – scenario A considers a 32% increase in price and scenario B considers a 62% increase in price. However, there are no discussions to justify the choice of the different scenarios. If it is what the government of Vietnam intend to do then the authors need to clearly state this in the work. *Response- *We have added this to the Methods section.
7. In the abstract, it is stated that Vietnam has over 15 million male smokers but in the study population section, the authors argued that their focus is on male smokers aged 15 years and older, because males comprised the vast majority of cigarette smokers in Vietnam (about 12.1 million out of 12.4 million overall). There is not information about the 2.9 million of the 15 million. What do the smoke? Or are they less than 15 years. This need to be made clear. *Response- *We have now corrected this. A total of about 15.2 million men smoked tobacco in Vietnam in 2015: about 12.1 million (out of 12.4 million cigarette smokers) are males (or 98% of total cigarette smokers); about 3.1 million men smoked hand-rolled tobacco, traditional bamboo waterpipe, shisha waterpipe, pipe, cigars/cheroots/cigarillos, and other forms of smoking tobacco.

# Peer review: Reviewer response for version 1

**DOI:** 10.21956/gatesopenres.14178.r27738

**Published:** 2019-10-03

**Authors:** Guillermo Paraje

**Affiliations:** 1 Business School, Universidad Adolfo Ibáñez, Santiago, Chile

There are no doubts, at least to me, that this type of analysis is extremely useful and pertinent to advancing policies on tobacco control. Extended Cost-Effectiveness (ECE) analyses are to this effort what General Equilibrium models are to the understanding of how an economy works in Economics. The fact that tobacco taxes, the single most effective policy to reduce tobacco consumption, have benefits that include reduce the financial burden that tobacco-related illnesses may entail for different population groups. The name Extended Cost-Effectiveness is misleading, as tobacco taxes do not have any cost involved if used to correct the externalities caused by tobacco consumption. These taxes do not have the usual efficiency losses involved in taxation, but rather an efficiency gain. Hence, the “cost” part in the name is far from being clear. At any rate, it is not something that can be blamed on the authors of the article.

 The article applies this methodology on Vietnam. The article uses ECE to assess the impact that two alternative proposals for increasing tobacco taxes would have on averted treatment costs, life-years gained and males avoiding catastrophic health expenditures and extreme poverty. The analyses focus on males, as female smoking rates are low, and by income quintiles to assess the distributional impacts of such policies. The conclusions of the article are that, under both tax increase scenarios males in the poorest quintile would benefit far more than those in the top quintile, both in terms of life-years gained and treatment costs averted.

 Overall, the results are worth what the assumptions in the analyses are worth and, though I believe findings are very important, they can be improved and/or made more robust (as the authors acknowledge when mentioning the limitations of the study). In this sense, the sensitivity analyses considered in the article can be improved. The key assumptions made on price elasticities for the different quintiles (page 5) are not well justified. They cite a previous study that estimated price elasticities for Vietnam, though such a study estimated elasticities for the bottom 40% and top 40% of households and in this study those figures are used for the bottom 20% and top 20% of them. It is highly likely to be true that poorer individuals have demands that are more price sensitive, as the evidence shows for many countries and also for Vietnam, but it would be convenient to reinforce the results with a good sensitivity analysis on elasticities.

 One way to do this that the authors may consider is, for a given value of the price elasticity for the top quintile, to determine the price elasticity value for the bottom quintile, for which the differential gains in health and costs averted disappear. In other words, to determine the elasticity for the bottom 20%, for which the “First: fifth ratios” in Tables 3 and 4 tend to one. My intuition is that such a parameter would be have an absurd value, given the existing evidence. If I am correct, that would be a definitive proof that for Vietnam tobacco taxes do have positive impacts on total population (unquestionable because tobacco demand slopes are negative), but especially for poorer individuals.

Some specific comments:

1. In the first paragraph of the introduction, when giving the number of smokers, it would be useful to have the month-prevalence. In fact, it is said what the target prevalence would be in 2020 but the current prevalence is never mentioned.
2. The tax structure outlined in Table 1 is not explained well. First, it is not clear whether the 10% VAT rate, for instance, is applied on all goods or on a subset of goods. Second, the tax base for the SCT is factory price, which raises the question of who determine the factory price (is it the manufacturer or the tax authority, as done in certain countries); and how realistic is the factory price, as it could be a mechanism to decrease taxes paid. To say that SCT rate on cigarettes is 75% sounds quite impressive, though it is far less impressive if the tax base is determined by the manufacturer and artificially low. This is crucial for the simulations. **If the tax base is determined by the manufacturers, how did the authors determine the tax bases to simulate the increase in the ad-valorem tax? **On page 5 they use a “current factory price of VND 8,028. **Where does that value come from?**
3. Though it is mentioned that a specific excise is imposed on tobacco, there is no explanation or description of values, taxable units, indexation mechanism, etc.
4. In the first paragraph of page 3, it is said that “The lower tax rates on cigarettes manufactured with domestic raw materials favoured domestic brands and encouraged per adult consumption of cigarettes.” I believe this is misleading: what favors consumption is not low taxes but low prices and/or affordable cigarettes. **Can the authors say something about these two variables and their evolution?**
5. On the same page, it is first said that “VAT is imposed on the added value of goods or services arising during the process from manufacturing up through retail sales”, and then that for VAT “the tax base is factory price plus the excise tax”. I believe that the two definitions cannot be true: one of them is incorrect, as there are stages in distribution and sale that, according to the first definition should be taxed, but according to the second definition would not be taxed. **Which is it?**
6. On the same page it is said that the TCF receives a contribution of 2% of “taxable price of all cigarette packs”. **Which is the taxable price?**
7. The authors put the smoking prevalence in the first paragraph of results. That, as suggested, should be moved to the first paragraph of the article.
8. On page 10 it is said that “One of the criticisms of increased tobacco excise taxes is that it increases smuggling. However, in Vietnam, cigarettes are smuggled into the country mainly to avoid import tax or due to the fact that smokers prefer well-known, illicit brands, both of which are not affected by the level of excise taxes.” I do not understand this sentence. **Does it mean that smuggled cigarettes only evade import taxes? Do they pay the tobacco taxes?** If that is the case, it should be stated explicitly. If, as I suspect, smuggled cigarettes do not pay tobacco taxes, the sentence is wrong and should be changed and a brief discussion on the relationship between tobacco taxes and smuggling in Vietnam could be provided.
9. In page 10 it is said that “Variation in price responsiveness has been reported in Vietnam, as recently reviewed by Fuchs and colleagues”. The analysis conducted in the referred document is quite unclear and, though this is not the place to discuss it, may have serious methodological limitations (e.g., endogeneity). It may also underestimate price elasticity as it seems to consider only the effect of price on intensity of smoking (intensive margin) and not the effect of price on the decision to smoke (extensive margin). Given these limitations I would suggest, again, the sensitivity analysis I propose above.

Is the work clearly and accurately presented and does it cite the current literature?

Yes

If applicable, is the statistical analysis and its interpretation appropriate?

Yes

Are all the source data underlying the results available to ensure full reproducibility?

Yes

Is the study design appropriate and is the work technically sound?

Partly

Are the conclusions drawn adequately supported by the results?

Partly

Are sufficient details of methods and analysis provided to allow replication by others?

Partly

Reviewer Expertise:

Economics; Development Economics; Health Economics

I confirm that I have read this submission and believe that I have an appropriate level of expertise to confirm that it is of an acceptable scientific standard, however I have significant reservations, as outlined above.

# Peer review: Reviewer response for version 1

**DOI:** 10.21956/gatesopenres.14178.r27684

**Published:** 2019-09-10

**Authors:** Nigar Nargis

**Affiliations:** 1 Economic and Health Policy Research, American Cancer Society, Washington, DC, USA

**Summary**

 Based on an extended cost-effectiveness analysis, this paper undertook a deterministic estimation of the effects of cigarette tax-induced price increase on smoking, public health, tax revenue, health expenditure and poverty status of households classified by five income groups from the poorest to the richest. The authors of the paper have extensively used this well-recognized methodology in previous research. It would make a valuable contribution to the advancement of the tobacco tax policy proposal under consideration in Vietnam by showing that tax and price increase do not only reduce smoking and improve public health, it can result in disproportionately larger health and financial gain to the poor and improvement in health and economic equity.

 While the paper has its merit, it requires major revisions to improve methods and reporting of results for it to be indexable. I have made very specific comments and recommendations separately for each section below. There are some typographical and grammatical errors that I have left to be taken care of at the final stage of production.

**Abstract**

1. Insert 'gained' after 'life-years' in the first sentence of the Results section.
2. The Conclusions section can be strengthened by rephrasing the final statement: 'Thus, tobacco taxes are an effective way to improve health, reduce poverty and improve health and economic equity'. Because the results focus on disproportionately larger health and financial gain and disproportionately smaller burden of revenue for the poorest. Unfortunately, the authors forgot to mention this conclusion altogether in the Conclusions section of the manuscript, which is very different from the conclusions stated in the Abstract. Please make the conclusions in the Abstract and in the main text consistent.

 
**Introduction** 

1. How many smokers were there according to GATS 2015? Even though prevalence decreased, number of smokers might have increased or remained the same due to population growth. It is advisable to show the comparison in terms of the number of smokers in addition to smoking prevalence in the opening paragraph. Because the results of the tax increase are reported in head counts (number of deaths avoided, number of people avoiding catastrophic health expenditure, number of people avoiding falling into extreme poverty) derived from the number of smokers who would quit.
2. Please add the following reference to show that the reduction in smoking has not been as high as expected based on comparative analysis from GATS 2010 and 2015: Minh HV, Giang KB, Ngoc NB et al. Prevalence of tobacco smoking in Vietnam: findings from the Global Adult Tobacco Survey 2015 ^1^.
3. The last sentence in the second paragraph states: “Also, effective tobacco taxation may contribute significantly to state budgets, if increasing revenue growth outweighs the percentage decline in consumption of tobacco products.” This statement needs some clarity. The fact that revenue increases in response to tax increases is attributable to inelastic demand for tobacco products. Inelastic demand means that the percentage change in consumption is lower than the percentage change in price and as a result consumers' expenditure on tobacco product increases. The price increase may lead to revenue gain if it is induced by tax increase. But there are other reasons for price increase in which case revenue may decrease. So 'tax-induce price increase' should be underscored.
4. In the sub-section “Taxation system on tobacco products in Vietnam”, in the first sentence of the second paragraph, it suffices to mention ‘tobacco product tax rates’ instead of ‘cigarette and tobacco product tax rates’. Restate the sentence as: Immediately after the introduction of the SCT, tobacco product tax rates were differentiated, creating a complex system that persisted until 2005.
5. In Table 1, please indicate the tax bases in the column heading for Value-added tax and Tariffs.
6. In Table 1, the cells in the bottom two rows with same tax rate can be merged like the previous rows to make the presentation consistent.
7. Page 4, first paragraph: How did the price of cigarettes made from imported raw materials compare to those made from domestic raw material? By stating that the smokers of the cigarettes made from imported raw materials had higher income, it is implied that these cigarettes are more expensive and hence affordable to higher income people. Is there any reference to support this presumption?
8. Page 4, second paragraph: The recommendation of specific tax is not originally from the World Bank document cited in the manuscript. The Article 6 Guidelines of WHO FCTC should be cited here.
9. Page 4, third paragraph: Please revise this statement as follows: For imported tobacco, the VAT taxable price is the import price at the border gate plus import duties plus excise tax. A uniform VAT rate applied to all tobacco products that remained constant.
10. In the sub-section “Current tobacco tax structure and rates in Vietnam”, please indicate if excise tax applies to imported tobacco products as well and whether at the same or at a different rate from domestic tobacco products.
11. Please elaborate CIF as Cost, Insurance and Freight, the first time it is used in the text in Page 4 in the sub-section “Current tobacco tax structure and rates in Vietnam”.
12. In the description of the Tobacco Control Fund in Page 4, please add the explanation of the ‘taxable price’ as follows: The TCF receives a compulsory contribution of 1% of the taxable price (pre-tax factory price for locally produced cigarettes and CIF value for imported cigarettes) of all cigarette packs produced locally or imported for local consumption beginning from May 2013.
13. In Page 4, the reference to the proposal of ‘mixed excise tax’ is not explained well in the second paragraph of the second column. Under mixed system, both options must have an ad valorem component and a specific component. The Scenarios A and B used for the analysis in this paper are both mixed. However, the way the proposal is described in this paragraph, it seems to me: Option 1: 75% ad valorem and VND 1,000 specific, which is a mixed system. Option 2: The ad valorem will be increased from 75% to 80% to 85% without any specific excise. This is pure ad valorem and not a mixed system. Also, what time frame ‘2020 onwards’ and ‘2021 onwards’ in the description of the proposal mean? Tax rates generally apply for a fiscal year. If I understood correctly, it should be described as: “increasing the ad valorem tax from 75% to 80% in 2020 and from 80% to 85% in 2021”? The phrase “2021 onwards” would imply that there would be no further increase in tax after 2021. Please describe the proposal more clearly to avoid confusion.
14. In Page 4, third paragraph in the second column, it is important to explain the following as the rationale of the analytical approach of the paper:

- What additional information the analysis by five income groups would provide that an aggregate level analysis would not.
- Why was it necessary to undertake this analysis in the context of Vietnam? For example, it might be the case that the government is hesitant to implement the tax proposal in fear of regressivity of the tax increase (that the increased tax burden might hit the poor most).

 
**Methods** 

1. Similar smoking prevalence by income quintiles and education levels is not the correct rationale for using education as a proxy measure of household income. Individual education can predict individual earning very well, but not necessarily household income. For education to be an appropriate proxy for income, the two variables must be strongly correlated regardless of the smoking status of individuals. Besides, income quintiles are based on per capita household income which is calculated as total household income divided by household size. Total household income can be a function of the education levels of household head and other earning members of the household. The use of individual educational status as a proxy of household income quintile in the youngest age group is subject to the caveat that upper secondary and college education are not completed at age 15 and individuals at this age do not necessarily earn income. Individual education after age 22, for example, can very well predict individual earning. Even then it does not necessarily predict household income status unless that individual is the head of the household or the principal earner of the family. For younger adults, it is usually their parents' education levels that would determine their household income status.  Researchers using GATS data construct wealth index to represent household income quintile as GATS does not collect household income data. I recommend constructing wealth index in place of individual education to proxy income in the present analysis. The income classification is at the heart of the analysis in this paper and hence needs to be carefully designed and interpreted. Please see the following article to consult on how to use the wealth index from GATS: Nargis N, Yong H-H, Driezen P, Mbulo L, Zhao L, Fong GT, et al. (2019) Socioeconomic patterns of smoking cessation behavior in low and middle-income countries: Emerging evidence from the Global Adult Tobacco Surveys and International Tobacco Control Surveys ^2^.
2. Page 5 Sub-section “Price effects on Smoking”: By ‘price elasticity of quitting’, I believe the authors meant the price elasticity of smoking prevalence which is generally taken as half of the total price elasticity (sum of the elasticity of smoking prevalence and smoking intensity). The price elasticity of prevalence reflects reduction in smoking both due to increased quitting and reduced initiation and transition to regular smoking habit. The authors indicated the part of the reduction in smoking due to quitting only. The price elasticity of quitting is positive because it represents the percentage increase in quitting probability in response to a given percentage increase in price. As such, price elasticity of quitting is not exactly interpretable as half of the price elasticity of cigarette demand. I recommend the authors look at Chapter 4 in Monograph 21: The Economics of Tobacco and Tobacco Control for detailed review of the studies that estimated price elasticity of quitting.
3. Page 5 Sub-section “Effect of cigarette price increase on life-years gained, disease costs, income poverty, and taxes paid”: The authors referred to GTEC and bypassed the description of the methods of analysis. While reading this section, the questions that came readily to my mind were: What are the coefficients that were used to translate the number of smokers who quit to number of deaths averted and number of life-years gained? How are catastrophic health expenditures and extreme poverty defined? I had to look at the BMJ paper to find the answers. The methods need to be presented in this paper as a stand-alone approach with the specifics and adjustments in parameters applied in the context of Vietnam. The GTEC can be referred to for the details.
4. Page 5 Sub-section “Effect of cigarette price increase on life-years gained, disease costs, income poverty, and taxes paid”: Please describe the treatment cost. Is it out-of-pocket expenditure only that covers medication, doctor's fee, hospitalization, etc? Does it also include government's expenditure? In what form is this variable available in the Statistics Yearbook--per patient/per visit for health care utilization, monthly/annual etc? A summary table of the treatment cost variables by the four disease categories would be informative.
5. Page 5 Sub-section “Effect of cigarette price increase on life-years gained, disease costs, income poverty, and taxes paid”: Conversion to international dollars is necessary for multi-country comparison. In this single-country paper, analysis in local currency and conversion into USD would be enough. That said, there is no harm in conversion to international dollars if it is done correctly. The following steps are required to do this conversion:

- First adjust prices/costs for inflation to express in base year constant prices (e.g. 2017 constant prices);
- Then divide the inflation-adjusted prices/costs by the purchasing power parity conversion factor of the base year (e.g. of 2017) to convert to inflation-adjusted international dollars. The description here says that Step 2 was done first and then Step 1. Please confirm if it was done in the right sequence.

 
**Results** 

1. Page 5 Sub-section “Cigarette smoking among males in Vietnam”: How do the income groups correspond to the education groups? For example, were the illiterate individuals coded as the poorest and those with college education or above coded as the top income group? It needs to be specified earlier in the Methods section.
2. Page 5 Sub-section “Cigarette smoking among males in Vietnam”: All tables present the income groups as first, second, third, fourth and fifth. Are those groups renamed as low, lower-middle, middle etc. income groups as written in the first paragraph of this section? Please maintain consistency in labeling the income groups throughout the manuscript.
3. Are the top and bottom income groups labeled correctly in the horizontal axes in Figure 1? The bottom income group seems to have lower gains than the top income group while the description says the opposite. The numbers 1 to 5 representing income groups seem correct, but the labels 'Top' and 'Bottom' were flipped.
4. What do the negative values of additional revenue collected in income groups 1 and 2 indicate in Figure 1 (d)? It is important to report the base revenue levels for each income group to enable one to interpret the negative values. For example, if the baseline revenue level collected from Income Group 1 was less than 3,000 billion VND, the additional revenue of -3,000 billion VND in Figure 1 (d) would imply that the after-tax increase revenue collection from Group 1 is negative. It means that smokers in the bottom income group would be subsidized instead of paying taxes.

**Discussion**

1. The Discussion section missed the discussion of the major finding of the paper that the poorest gain most from the tax increase which helps gain health and economic equality. Both the Discussion and the Conclusion sections need to be aligned to highlight this very important finding.
2. The GTEC paper included Vietnam among the 13 middle income countries. Please elaborate what this paper adds to the analysis or findings over those of the GTEC paper. Why was it necessary to publish a country-specific paper for Vietnam while it was already available in a multi-country paper? 
3. Does the study by Jha and colleagues (2019) show increase in revenue? Please indicate it because the leading statement in this paragraph makes the point that revenue increases despite decrease in consumption and you are providing supporting evidence from different countries. The reference to illegal cigarette sales is cannot be put in context here unless the study also shows that smoking decreased while revenue increased event after accounting for illegal cigarette sales.
4. The statements below in the Discussion section are not substantiated or do not directly follow from the results of the paper. They are rather speculative. 

- “Compared to Thailand and the Philippines, Vietnam has the highest annual consumption of 3,900 million packs but collects only about one-third of the total tobacco tax collected in Thailand or the Philippines due to the current low tax rate.” Without detailed information on the number of cigarettes packs consumed in the three countries and the comparison of tax rates as percentage of retail price, this claim is not warranted. I recommend taking this statement out unless the authors can cite a source with this comparison.
- “Although the tax revenue itself would not provide enough to meet the financial needs of universal health coverage, it would make a significant contribution.”
- Is any estimate of the cost of financing universal health coverage in Vietnam available? What percentage of this cost can be covered by the increased revenue?

I recommend removing both these statements.

**Conclusions**

1. The Conclusion section should emphasize the fact that the poorest gain most from tax increase and that this finding dispels the myth that tobacco tax increase is regressive.
2. The policy implication written in the conclusions section to qualify large increases in price particularly those that focus on narrowing the gap between the least and most expensive cigarettes does not follow from the finding of this paper. The analysis was done using the price of the most sold brand. A more nuanced analysis with differential prices and consumption by brands at different price tiers is needed to draw this conclusion. Please omit this statement. The Conclusion section must cover what this study found in terms of the overall effect of tax increase on smoking, public health and tax revenue as well as greater public health gain for the poor.

Is the work clearly and accurately presented and does it cite the current literature?

Partly

If applicable, is the statistical analysis and its interpretation appropriate?

Not applicable

Are all the source data underlying the results available to ensure full reproducibility?

Yes

Is the study design appropriate and is the work technically sound?

Partly

Are the conclusions drawn adequately supported by the results?

Partly

Are sufficient details of methods and analysis provided to allow replication by others?

No

Reviewer Expertise:

Economics of tobacco and tobacco control

I confirm that I have read this submission and believe that I have an appropriate level of expertise to confirm that it is of an acceptable scientific standard, however I have significant reservations, as outlined above.

## References

[1] : Prevalence of tobacco smoking in Vietnam: findings from the Global Adult Tobacco Survey 2015. *Int J Public Health*.2017;62(Suppl 1) : 10.1007/s00038-017-0955-8 121-129 10.1007/s00038-017-0955-8 28229183

[2] : Socioeconomic patterns of smoking cessation behavior in low and middle-income countries: Emerging evidence from the Global Adult Tobacco Surveys and International Tobacco Control Surveys. *PLoS One*.2019;14(9) : 10.1371/journal.pone.0220223 e0220223 10.1371/journal.pone.0220223 31490958PMC6730869
